# A Kinetic Model-Driven Techno-Economic Analysis of Plastic Pyrolysis: Linking Process Dynamics to Economic Viability

**DOI:** 10.1021/acs.iecr.6c00693

**Published:** 2026-05-27

**Authors:** Farhad Zaker Hosseiny, Rui Shi

**Affiliations:** † Department of Chemical Engineering, 8082 Pennsylvania State University, University Park, Pennsylvania 16802, United States; ‡ Institute of Energy and the Environment, 311285 The Pennsylvania State University, University Park, Pennsylvania 16802, United States

## Abstract

This study employs a kinetic model integrated into Aspen Plus to predict pyrolysis product distribution under various conditions. A techno-economic assessment calculated the minimum selling price (MSP) of pyrolysis oil under different operating conditions for the baseline capacity of 100 kta, and across eight processing capacities ranging from 30 to 150 kta. The lowest MSP under the baseline capacity is estimated at $420/ton, which is 33% lower than the 2023 average US crude oil price ($74.6/bbl, equivalent to $634/ton based on the density of pyrolysis oil). Under Monte Carlo simulation, accounting for variability in key economic and technical parameters, the mean MSP is estimated at $1137/ton. The economic viability depends on feedstock price remaining below $320/ton, defining the break-even feedstock price threshold. Sensitivity analysis further identifies capital investment and transportation cost as key economic drivers. Capacities beyond 90 kta show limited economies of scale benefits. Reducing product storage time cuts capital costs by 7% but raises operational risk. Uncertainty analysis suggests the economic feasibility of pyrolysis oil is unlikely to compete with crude oil without policy incentives.

## Graphical abstract

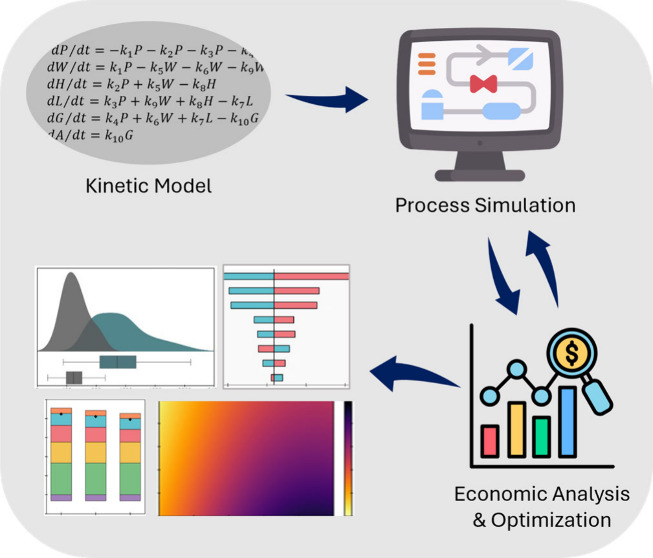

## 1 Introduction

With the continued rise in global plastic production and the short lifespan of plastics, particularly in the packaging sector, plastic waste generation now exceeds 300 million tons per year worldwide (the United States alone generated 36 M tons in 2018). Of this, approximately 79% is landfilled, 12% is incinerated, and less than 9% is recycled as of 2018 in the United States.^1–3^ The primary drawback of conventional methods such as landfilling for managing plastic waste is their lack of profitability, as they only impose additional costs on governments. This economic burden often leads to mismanagement, particularly in developing countries, ultimately resulting in the accumulation of plastic waste in oceans and ecosystems. Such accumulation poses significant threats to ecosystem, marine life, and human health.^4–7^

Currently, mechanical recycling is the most widely used method for plastic recycling on an industrial scale. However, its application is limited to certain types of plastics, such as high-density polyethylene and polyethylene terephthalate (PET) bottles, while other plastic types face significant challenges in practical implementation. In addition to issues related to contaminants and impurities, thermo-mechanical degradation occurring during the recycling process also negatively affects the mechanical properties and quality of the recycled plastics.^8–15^ As a result, mechanical recycling is unable to process the entire volume of plastic waste collected at materials recovery facilities (MRFs). To address these limitations, researchers are exploring various chemical and thermal methods, with pyrolysis emerging as the most extensively studied technique.^15,16^ Several companies, including Agilyx, Pyrowave, Encina, Recenso GmbH, and LyondellBasell, have established plastic recycling facilities based on pyrolysis at the pilot or early commercial stage in various regions worldwide.^17^ However, broader application still remains limited due to high capital costs, inconsistent feedstock quality, challenges in final product quality control and subsequent upgrading steps, and regulatory approval.^18,19^

Since these advanced methods are still in development, system analysis is essential to ensure economic viability across varying and complex conditions. Early studies demonstrated that advanced recycling of plastics can be economically viable, with payback periods ranging from 24 to less than 3 years depending on various factors such as processing capacity, taxation, tipping fees, operational hours, production rates, and feedstock calorific value.^20–22^ Later studies shifted focus to comparing the various recycling methods currently under development. Pyrolysis has often emerged as the most economically viable option among existing and emerging recycling methods. However, inconsistent operating conditions, system boundaries, and economic assumptions can lead to conflicting results and conclusions among different studies. For instance, one study deemed gasification unprofitable with a negative internal rate of return (IRR), while another found it highly profitable with an IRR of 17%.^23,24^ An evaluation of mechanical, chemical, and combined recycling of mixed plastics waste suggested that combined recycling is superior to individual methods in terms of costs. The virgin material substitution ratio for mechanically recycled plastics were shown to have significant impact on overall sustainability.^25^ As with any energy-intensive process, proper heat integration can improve the economic viability of plastic pyrolysis, potentially reducing the minimum selling price of the main product by up to 4% ^26,27^ Multiproduct pyrolysis-based refinery is another process concept offering the processing flexibility and economic viability.^21,26,28^ Focusing on producing more valuable products, such as lubricants, could further enhance the profitability of the process.^29^ However, the profitability can be easily compromised by product yield, as the added revenue from multiple and more valuable products may not fully offset the increased capital and operational costs.^30,31^

Among various factors influencing profitability, feedstock price is consistently identified as a key factor influencing the economic viability of recycling plants in nearly all techno-economic studies. This factor alone can contribute approximately 50% to the minimum selling price of the final product.^27^ Sensitivity analysis demonstrates that even relatively minor fluctuations in the purchase cost of feedstock can lead to significant variations in the minimum selling price of the final product. The impact of this factor can be so dominant that a linear relationship can emerge between the feedstock price and the minimum selling price of the main product, as determined by discounted cash flow analysis.^32^ The assumed purchase cost of feedstock in various techno-economic analysis (TEA) studies varies considerably, ranging from zero to $600 per ton. This discrepancy is likely a major factor contributing to the inconsistency of TEA results across different studies. For example, while Fivga^22^ demonstrated that the overall costs associated with fuel production could be ten times lower than the market price of fuels (assuming no cost for feedstock), Yadav,^32^ assuming a feedstock cost of $600 per ton, found that the price of pyrolysis-derived naphtha could be more than four times higher than naphtha produced from crude oil. Consequently, feedstock price has become a focal point in determining the threshold necessary for ensuring the profitability of plastic recycling. Reported thresholds have varied widely, ranging from $200 to $700 per ton, primarily due to differences in economic assumptions, feedstock type, target products and byproducts, and processing capacity across studies.^26,27,32–34^

This literature review highlighted the influence of various market-driven and engineering factors on the economic viability of plastic recycling. While previous studies have extensively explored these factors, this study moves beyond static TEA evaluations by adopting a dynamic approach that examines how an economic viability metric interacts with reaction conditions. This study aims to address this gap by incorporating a kinetic model into the process simulation, which enables evaluating the economic sustainability of the process under a wide range of design and operating conditions. The dynamic nature of this approach enables a more agile and predictive assessment of how variables such as temperature and vapor residence time impact product yield, and consequently, economic performance of the system. Additionally, by incorporating uncertainty analysis under a Monte Carlo framework into our model, we can better evaluate the economic performance of the recycling process and its long-term viability under different market and operational scenarios. Ultimately, our approach provides a more comprehensive understanding of the trade-offs while facilitating the identification of reaction conditions that maximize profitability in thermo-chemical recycling of plastics.

## 2 Methods

### 2.1 Conceptual Design

The recycling facility in the baseline scenario in this study is designed to process 100 kilotons per annum (kta) of waste polypropylene through noncatalytic thermal pyrolysis, assuming 330 operational days per year. This baseline capacity exceeds that of most currently operating pyrolysis plants.^17^ However, few facilities currently under construction are designed with processing capacities around or greater than 100 kta, suggesting that such scales are plausible in the near term.^35,36^ Furthermore, the selected capacity is consistent with the range commonly adopted in techno-economic analyses of plastic recycling technologies, thereby enabling meaningful comparison with existing studies. The feedstock consists of postconsumer polypropylene (PP) bales, which are primarily composed of PP and are sourced from MRFs.

The process is divided into four main sections (Figure 1). The process begins in the A100 Pre-processing area, where incoming plastic bales undergo mechanical size reduction. Rigid plastics are crushed into uniformly sized flakes of 2–3 mm using a grinder, producing material suitable for subsequent thermal processing. These flakes are then fed into the A200 Pyrolysis unit, where they undergo thermal decomposition in an oxygen-free environment, breaking down into lighter hydrocarbons. The resulting vapors are directed to the A300 Separation area, where they are cooled in heat exchangers using cooling water and separated into liquid and gaseous fractions via flash drums. In the A400 Storage area, the separated products are stabilized and stored under appropriate conditions: the pyrolysis gas byproduct is compressed to 13 bar, cooled to 30 °C, and stored as a pressurized liquid in cylindrical tanks, while the pyrolysis oil product is cooled to 30 °C and stored at atmospheric pressure in floating roof storage tanks.

**Figure 1:**
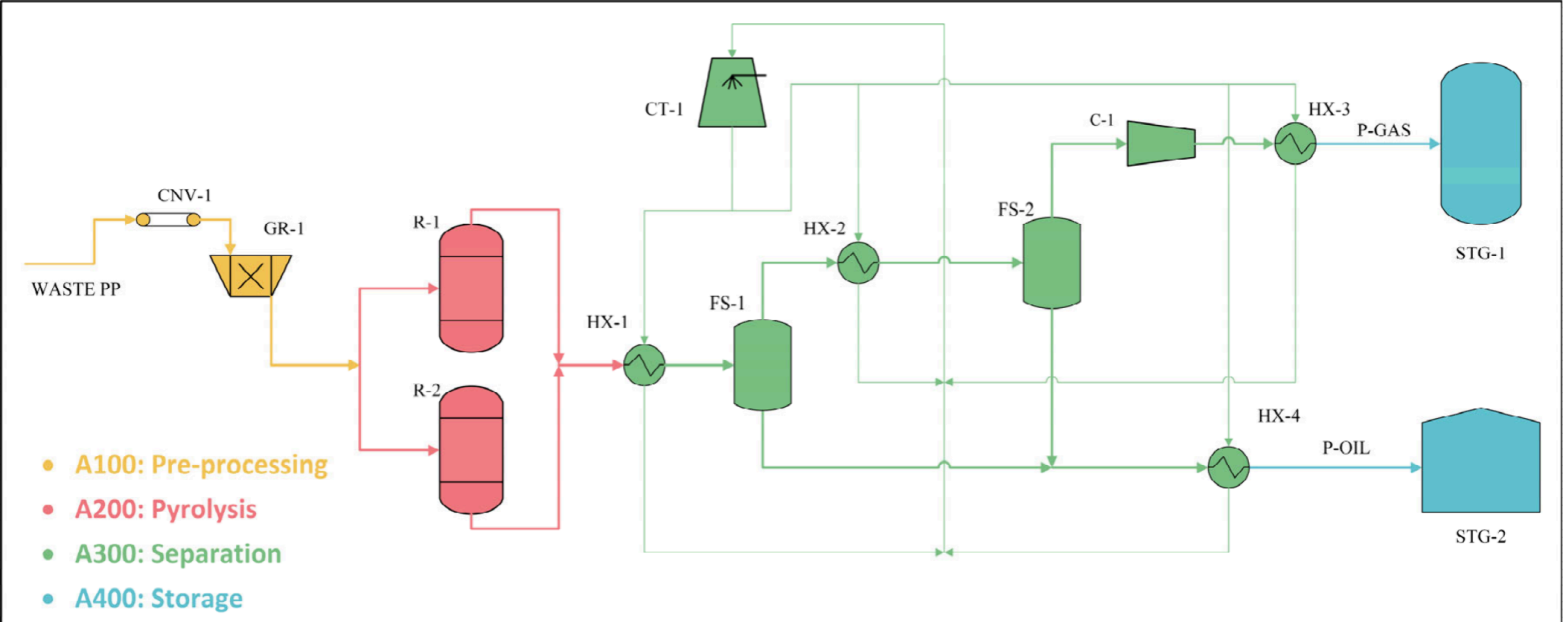
Schematic diagram of the plastics recycling process via pyrolysis.

The process generates two primary outputs: (1) pyrolysis oil (P-Oil), as the main product, and (2) combustible pyrolysis gas (P-Gas), as the byproduct. For techno-economic evaluation, the P-Gas is assumed to be a substitute for natural gas for heat and energy generation purposes, and P-Oil is assumed to be a replacement for crude oil in refinery systems. Among potential reference products (e.g., naphtha), crude oil was selected because both crude oil and P-Oil require extensive refining and upgrading prior to conversion into fuels or petrochemical intermediates. Furthermore, the lower heating value (LHV) of P-Oil observed under the different reaction conditions in this study (38–45 MJ/kg) was comparable to the reported LHV of crude oil (43.4 MJ/kg).^37,38^ In contrast, assuming direct substitution with naphtha would neglect the downstream refining and upgrading steps, overestimate the substitution potential, and apply a higher benchmark market value, thereby resulting in an overly optimiztic economic assessment.

Despite these similarities, notable differences also exist between P-Oil and crude oil in terms of chemical composition and impurity content. P-Oil derived from the pyrolysis of polyolefins primarily comprises olefinic compounds, with concentrations reaching up to 50%.^39–41^ In contrast, crude oil is primarily composed of paraffins, naphthenes (cycloparaffins), and aromatic compounds, with olefins being virtually absent in its natural state and only formed during downstream refining processes.^42,43^ Additionally, plastic-derived pyrolysis oil generally exhibits substantially higher levels of organic and metal contaminants compared to crude oil,^19^ necessitating additional upgrading and controlled dilution prior to full integration into conventional refinery operations.

In this study, the system boundary is defined at the output of the pyrolysis oil refinery stage, excluding subsequent upgrading steps such as hydrotreatment, distillation, or blending that would be required to meet transportation fuel specifications. This boundary reflects current industrial practice, where most pyrolysis companies produce and sell stabilized pyrolysis oil without further upgrading. Oil upgrading technologies have been commercially mature and well established throughout the petroleum and chemical industries over the past several decades. Continuous advancements over the past several decades have improved their energy efficiency, reliability, and cost-effectiveness, resulting in substantial reductions in both capital expenditures (CAPEX) and operational costs (OPEX). Therefore, it is more practical and economically favorable to utilize existing petroleum refining facilities for upgrading, rather than building new small-scale units at plastic recycling sites. Moreover, given the challenges in collecting and transporting waste plastics, decentralized and modular pyrolysis facilities are increasingly recognized as scalable, cost-effective solutions for localized plastic waste management.^44,45^ While the baseline scenario assumes a processing capacity of 100 kta, the study also explores lower-capacity scenarios representing decentralized (distributed or modular) configurations. Including complex upgrading units in these smaller-scale cases would be unrealistic and economically infeasible. Therefore, by limiting the system boundary to the production of stabilized pyrolysis oil using unsophisticated separation units, this study ensures consistency across scales and focuses on the core thermal conversion processes applicable in both centralized and distributed setups.

### 2.2 Integration of Kinetic Modeling and Dynamic Simulation in ASPEN Plus

To capture the dynamic behavior of the pyrolysis reactions, the process simulation was integrated with a mechanistic, lumped-type kinetic model. This coupling enables more robust and detailed predictions of reaction performance and overall process economics under a range of operating conditions. The kinetic model used in this study includes six lumped species and ten reactions, all of which are assumed to follow first-order, irreversible kinetics.^46^ Corrections were required to reproduce the original model’s outputs due to inconsistencies in the published kinetic parameters; these corrections are documented in Table S3 in the SI. It should be noted that the kinetic model has limitations in predicting product distributions at larger scales. First, the model was originally developed based on data from a micropyrolysis reactor, where heat and mass transfer resistances are minimal. In contrast, industrial-scale systems exhibit significantly more complex transport phenomena, which can influence reaction pathways and product yields. Second, the model assumes that feedstock impurities have a negligible effect on the pyrolysis process. However, recent studies have demonstrated that even trace levels of contamination can significantly impact product distributions and yields.^13^ The contamination level (the amount of impurities present in PP bales) in postconsumer PP sourced from MRFs can be as high as 8% by weight.^47^

Aspen Plus with Peng–Robinson thermodynamic model is used to simulate the process and perform heat/mass balance calculations. The product names used in the process simulator were aligned with the lumped species defined in the original kinetic model: Gas, Light Oil, Heavy Oil, Wax, and Aromatics. Waste PP was modeled as a solid. The Gas fraction, which in the kinetic model represents light hydrocarbons (C_1_–C_4_), in our simulation was modeled by propene (C_3_H_6_) as the representative species, as studies already shown that propene is the dominant component of the gas fraction in waste PP pyrolysis.^41,48,49^ “Light Oil”, “Heavy Oil”, “Wax”, and “Aromatics” were represented by pseudocomponents with a carbon number equal to the average carbon number associated with each lumped species in the kinetic model. The process flow diagram in process simulator is provided in the Figure S1 in the SI.

The process utilities considered within the battery limits include: (1) electricity, used to power pumps, grinders, and compressors; (2) cooling water, with an inlet temperature of 25 °C and an outlet temperature of 35 °C, serving as the cooling medium in separation units; and (3) natural gas, which supplies the thermal energy required for polymer decomposition in the pyrolysis reactor. The consumption rates for each utility were determined through process simulation, based on mass and energy balance calculations and user-defined operational criteria specific to each utility. The approach temperature in the cooling system was assumed to be 5 °C. LHV of natural gas was assumed to be 47.14 MJ/kg,^50^ and the thermal efficiency of the pyrolysis furnace was considered to be 70%, representing typical performance for industrial-scale systems.^51,52^

The process flowsheet in the process simulator includes two calculator blocks. The first block solves a system of six ordinary differential equations (ODEs) in the kinetic model, allowing the prediction of product distribution at different temperature levels and vapor residence times in the reactor. The ODEs are solved numerically using fourth order Runge–Kutta method due to its accuracy, robustness, adaptability, and ease of implementation.^53^ Reactor temperature (*T*), vapor residence time (VRT), and Arrhenius parameters of reaction constants (*A* and *E*_i_) are the primary input parameters for the calculator block, which estimates the mass fractions of lumped species as outputs. These calculated mass fractions are subsequently passed to the yield reactor, where they define the product distribution. Variation in operating conditions (*T* and VRT) directly influence the pyrolysis reaction kinetics and, consequently, the composition of the reactor effluent. The resulting changes in effluent temperature and composition directly impact the thermal requirements (heat duties) of both the pyrolysis reactor and the downstream separation units, thereby influencing the utility consumptions across the process. Tables S1 and S2 in the SI present the stream and utility summaries for a single representative reaction condition.

### 2.3 Engineering Economics

We use minimum selling price (MSP) as the economic indicator of this study, determined through discounted cash-flow (DCF) analysis. This analysis is performed using both Aspen Plus, via the second calculator block, and Excel spreadsheets. Equipment sizing and cost estimation (total capital investment and fixed operational costs) were determined using Aspen Process Economic Analyzer (APEA). Variable operating costs were calculated manually. Most costs and material prices (except for some fixed operating costs components) were based on 2023 average values in the United States or adjusted to 2023 dollars using cost indices. A full summary of economic assumptions is provided in Table S4 in the SI to ensure transparency and consistency in the TEA. In this study, P-Gas corresponds to the gas fraction in the kinetic model, while P-Oil is defined as the sum of the remaining product fractions, namely light oil, heavy oil, wax, and aromatics.

### 2.4 Operating Conditions and Economic Viability

The dynamic nature of this simulation (as a result of integrating the kinetic model into process simulator) facilitated a two-variable semiglobal sensitivity analysis in Aspen, enabling a systematic evaluation of how variations in reaction conditions (*T* and VRT) affect the MSP, the economic indicator for profitability in this analysis. By directly linking reaction conditions to a profitability indicator, this analysis clarifies the relationship between process parameters and economic performance and provides a quantitative basis for identifying the conditions that yield the lowest MSP (maximum profit). This is done through the second calculator block. In this block, total capital investment, fixed operational costs, tax rate, and feedstock acquisition and transportation expenses are fixed input parameters while the product and byproduct flow rates and utility consumptions are the variables which change with operating conditions. Although the tax rate is assumed to be constant, the total tax paid is proportional to the annual cash flow, which varies depending on product flow rates and utility consumption throughout the process. With all necessary components available for the cash flow analysis, the MSP is determined numerically using the iterative “modified secant ” method. This iterative approach offers an advantage over the conventional secant method, as it requires only a single initial guess (in contrast to the two initial estimates needed by the standard method).^54^ This enables the “modified secant ” method to be more efficient and practical for estimating MSP in techno-economic assessments where the objective function (net present value) is sensitive to multiple interacting variables. To verify the accuracy of the numerical method and its correct FORTRAN implementation (the compatible language for user-defined calculations and codes in Aspen Plus), the MSP calculations were independently validated in Microsoft Excel using its built-in solver under identical techno-economic assumptions. The results were identical across both platforms, confirming the correctness and reliability of the implementation. The results of this analysis defined the reaction conditions for the base-case scenario in all subsequent analyses.

### 2.5 Sensitivity and Scenario Analyses

In addition to the sensitivity analysis performed in the process simulator, a one-at-a-time single-variable sensitivity analysis was conducted on economic parameters to monitor the variation of MSP. The variation extant in feedstock price (ranging from $113/ton to $184/ton) was selected based on average low and high prices in the US during 2023 collected by *RecyclingMarkets*.^47^ While this range captures temporal price variability at the national level, it does not distinguish the effects of feedstock grade, contamination level, or compliance with specification requirements on pricing. Consequently, the price variation is treated solely as a function of supply–demand dynamics, representing a source of uncertainty and a limitation of the present analysis due to the lack of more detailed market data. A variation of ±20% was applied to the baseline estimates of transportation costs, total capital investment, and fixed operational costs, as these parameters are generally subject to moderate uncertainty.^29^ A slightly wider variation of ±25% was applied to utility costs and P-Gas sale price to account for their higher volatility. Overall, these ranges are consistent with values commonly reported in the literature,^22,31,32^ enabling meaningful comparison with previous studies. The variation in furnace heating efficiency was determined based on values reported in the literature^52^ and our engineering judgments. The tax rate was varied between 16% and 36%. All baseline assumptions and estimates, along with the associated methodologies and references, are provided in the SI.

The scenario analyses in this study explore multiple cases based on key design heuristics and decisions, specifically focusing on processing capacity and storage duration. Processing capacity impact profitability, largely through economies of scale, while storage duration influence supply chain flexibility by buffering fluctuations in feedstock availability and product demand. It should be noted that a detailed supply chain analysis is beyond the scope of this work. No scale-up heuristic rules (such as the commonly used six-tenths rule^55^) were applied in this analysis. Instead, for each capacity scenario, a distinct process flowsheet was developed in Aspen Plus, and the corresponding economic calculations were performed using APEA. This approach ensures consistency with the base case and allows for more accurate comparisons across scales. A total of eight capacity scenarios, ranging from 30 kta to 150 kta, were evaluated. Lower capacity scenarios are intended to reflect distributed or modular system configurations, whereas higher capacities represent centralized processing facilities. For the storage duration analysis, two additional cases, 14 and 21 days, were considered alongside the 28-day base case at a processing capacity of 100 kta. In all cases, equipment sizing and cost estimation were performed independently (using APEA) to improve the accuracy and reliability of the comparisons.

### 2.6 Uncertainty Analysis

Following the variation of MSP across different scenarios and the reaction conditions that minimize the MSP identified, a Monte Carlo simulation was conducted on the base case to assess the impact of parameter uncertainty on MSP. This simulation employed a spreadsheet-based framework developed in Excel, supported by custom-developed macros. The analysis accounted for uncertainty in 20 key techno-economic parameters. Unlike many existing works^56^ that rely on statistically efficient techniques such as “Latin Hypercube Sampling” to reduce computational burden, this study intentionally employed completely random sampling. This decision was made to prioritize the robustness and reliability of the uncertainty analysis over computational efficiency, as random sampling ensures a broader and less structured coverage of the parameter space. Consequently, a total of 27 000 trial runs were performed, which is well above the typical range of 2000–5000 runs in comparable analyses.^56,57^ To verify that the number of trial runs used in this study was sufficient for a robust analysis, an additional Monte Carlo simulation with 50 000 iterations was performed. The results from both simulations were identical, confirming the adequacy of the original run count. To improve the analytical accuracy of the analysis, the interdependencies among process variables were defined using mathematical correlations linking them to one another. For instance, natural gas consumption in the pyrolysis reactor was modeled as a function of the thermal energy demand, the LHV of natural gas, furnace heating efficiency, and annual operating days. Likewise, utilities such as cooling water usage and compressor power requirements in the separation section were correlated with pyrolysis oil yield (refer to Figure S3 in the SI for details). A complete list of the input parameters used in the uncertainty analysis, including their defined variation ranges, assigned probability distribution functions, and relevant literature references where applicable, is provided in Table S11 in the SI, ensuring transparency and reproducibility of the simulation approach.

## 3 Results and Discussion

### 3.1 Baseline TEA Results and Sensitivity Analysis

The total capital investment required to construct the plant at the baseline capacity of 100 kta is estimated at $46 million, comprising a fixed capital investment of $41.5 million and a total installed equipment cost of $19 million (2023 dollars, CEPCI adjusted), with working capital set at 10% of the fixed capital investment. Although this percentage may underrepresent actual working capital needs at smaller scales where fixed operational costs tend to be a larger fraction of total operational costs, adopting a uniform assumption avoids introducing variability that can impose additional uncertainty in the analysis and comparisons across different plant capacities. Detailed capital expenditure breakdowns are presented in Tables S5–S7 in the SI. The total annual operational cost is approximately $30 million. Raw material costs dominate the operating expenses (48.2%), followed by feedstock transportation (31.8%). Utilities, which include electricity, cooling water, and natural gas, accounts for less than 7% of OPEX. The remaining 13% is associated with expenses such as labor, maintenance, and other fixed operating costs essential for sustaining day-to-day plant operations (Figure 2A, also detailed in Table S6 in the SI). A discounted cash flow analysis yields a minimum selling price of P-Oil at $423.6/ton, which is below the 2023 average US crude oil price of $74.6/bbl (or $634/ton on mass basis).^58^

**Figure 2:**
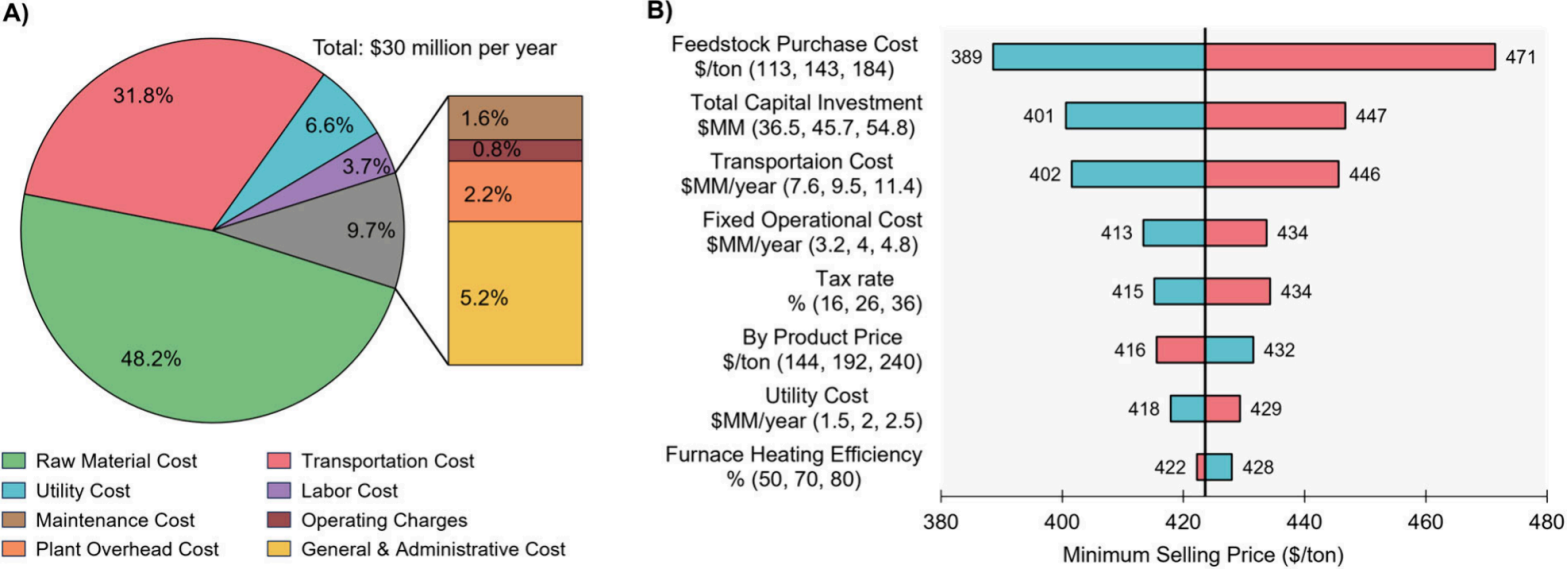
(A) Breakdown of operating cost for the baseline scenario at 100 kta processing capacity. (B) Single-variable sensitivity analysis showing the influence of key economic parameters on the minimum selling price of the P-Oil (*T* = 525 °C, VRT = 6 s).

Figure 2B shows the impact of key techno-economic parameters on MSP of the final product. The MSP is most sensitive to feedstock purchase cost, capital investment, and transportation cost, while utility cost and furnace heating efficiency have comparatively lower influence. Sensitivity analysis results highlight the critical role of feedstock and product logistics in overall economic performance. This is further supported by the fact that storage infrastructure alone accounts for 66% of the total equipment cost (Figure S2 in the SI), making it the largest contributor to capital expenditure. Between the two logistics components, the feedstock supply chain exerts a more substantial influence. Nonetheless, this balance could potentially shift, partially or entirely, if the system boundary were expanded to include product transportation costs, as the current MSPs are calculated at the facility gate. Variations in fixed operational cost, tax rate, byproduct price, and utility costs have a similar impact on minimum selling price and are minor compared to the first three parameters. The heating efficiency of the pyrolysis furnace had the least influence on economic outcomes. This limited impact stems from the simplicity of the kinetic model, which does not account for heat transfer effects on product distribution, despite evidence in the literature suggesting otherwise.^59^ In this study, furnace efficiency only affected natural gas consumption, a component of total utility costs, further diminishing its overall economic significance.

### 3.2 Impact of Operating Conditions on Profitability

Integration of the kinetic model enables understanding of the economic potential across various operating conditions. Figure 3A shows that generally lower temperature and higher residence time favors P-Oil formation. There is a slight increase in the oil content at higher temperature levels as extended residence time favors the aromatization reactions (gas fraction is converted to aromatics). The observed trend in MSP (Figure 3B) mirrors that of pyrolysis P-Oil yield, which can be attributed to two key factors: (1) higher P-Oil yield increases revenue, and (2) the same reaction condition also minimize utility demands (lower duties for heating, cooling, and P-Gas compression), lowering OPEX. The MSP of pyrolysis oil varies between $420/ton and $490/ton, depending on reaction conditions, indicating that choosing the right operating condition in the reactor could reduce the MSP by up to 20%. In this analysis, process equipment was sized based on the maximum energy and power requirements across the entire range of reaction conditions. Designing the equipment based on lower temperatures, with limited overdesign to ensure reliability, could lower the CAPEX and thereby further reduce the MSP.

**Figure 3:**
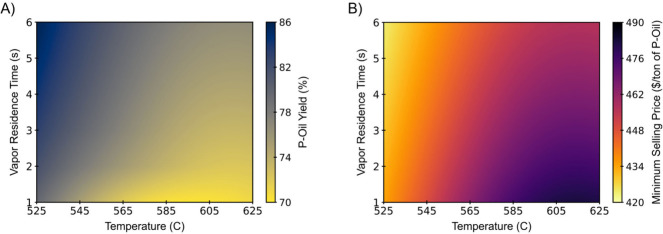
Impact of reactor temperature and vapor residence time on (A) P-Oil yield, and (B) minimum selling price of P-Oil. All results correspond to a plant capacity of 100 kta.

The lowest estimated MSP (at *T* = 525 °C and VRT = 6 s) in this analysis is 33% lower than the average US crude oil price in 2023 ($74.6/bbl).^58^ In fact, provided that the purchase cost of waste PP bales is less than $320/ton, the process is profitable. However, this assumption appears overly optimistic when we compare the price of waste Natural HDPE to waste PP in the same timeline (2023) and recognize how rising demand could drive prices even higher. For instance, if we assume the average purchase cost of feedstock is equivalent to that of waste Natural HDPE ($1053/ton^47^), the minimum selling price of pyrolysis would be $1480/ton, which is more than 2.3 times higher than the price of crude oil. Additional details on economic calculations are provided in Figure S2 and Tables S2 and S5–S8 in the SI.

### 3.3 Scenario Analyses

Informed by the sensitivity analysis, scenario analyses were conducted to quantify the effect of key operational and economic parameters on process performance. The scenarios examined include (1) product storage duration, (2) process downtime (3) the impact of feedstock cost and (4) plant scale. These analyses were performed to assess their respective effects on the minimum selling price and overall economic feasibility.

#### 3.3.1 Impact of Product Storage Duration and Process Downtime on System Economics

Given that the product storage area represents the most cost-intensive section of the process when considering only directly attributable costs (installed equipment costs and utility costs; Figure S2 in the SI), we evaluated the impact of reduced storage durations on the MSP. The analysis included storage durations of 14 and 21 days in addition to the 28-day base case. As shown in Figure 4, reducing storage duration can lower the minimum selling price from $423.6/ton to $395.1/ton in the 14-day scenario, representing approximately a 7% reduction (more details in Table S9 in the SI). Across all storage scenarios, the contribution of individual cost components to the MSP remains largely consistent, except for CAPEX, which varies as expected. The purchase and transportation costs consistently constitute the largest portions of the MSP.

**Figure 4:**
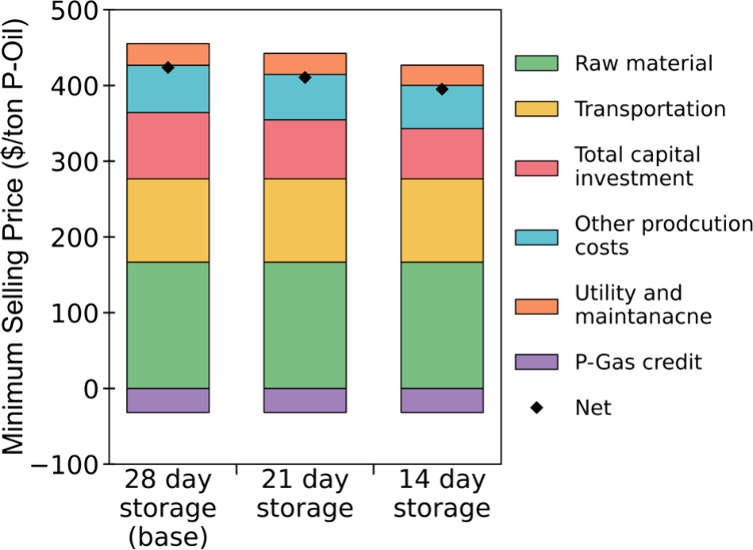
Variation in P-Oil minimum selling price with storage duration. Colored segments represent individual contributors, and the markers indicate the net value of minimum selling price. All results correspond to a plant capacity of 100 kta.

It is important to note that storage duration largely depends on logistics. Typically, the distance between the plant and the customer (destination) directly influences storage needs. Here, we assume that refineries are the primary customers for these products. Thus, interpreting a shorter storage duration as closer proximity to refineries would likely increase feedstock transportation distances and costs, factors not accounted for in this analysis. Therefore, while reducing product storage duration may help reduce MSP, operational logistics could impose additional complexities, such as unexpected shutdowns, that influence the overall economic outcome. Extended downtimes, whether due to maintenance or supply chain issues, can affect process economics, with the MSP of pyrolysis oil rising from $419/ton to $451/ton (equal to $0.51/ton increase for each additional day of shutdown) as downtime increases from 25 to 85 days (Figure S5 in the SI).

#### 3.3.2 Impact of Feedstock Cost and Processing Capacity

Figure 5 illustrates the impact of feedstock price and processing capacity on MSP of P-Oil. Scenarios were developed by varying processing capacities from 30 to 150 kta and waste PP prices between $0 and $600 per ton. As processing capacity increases, sourcing sufficient quantities of waste PP would generally require input from a broader geographic area, given that the majority of MRFs in US handle less than 100 kta of throughput,^60^ and PP comprise less than 1 wt % of curbside collections.^61^ Accordingly, for modular facilities with capacities below 50 kta, a transportation distance of 80 miles was assumed, whereas a distance of 200 miles was applied for centralized facilities with capacities exceeding 50 kta. These values are consistent with industry survey data reported by Argonne National Laboratory.^62^ For the 50 kta scenario, an intermediate distance of 140 miles was adopted, as the survey does not explicitly classify this capacity as either modular or centralized. This assumption also provides a relatively smooth transition between the two regimes, avoiding an abrupt change in transportation distance at the capacity threshold.

**Figure 5:**
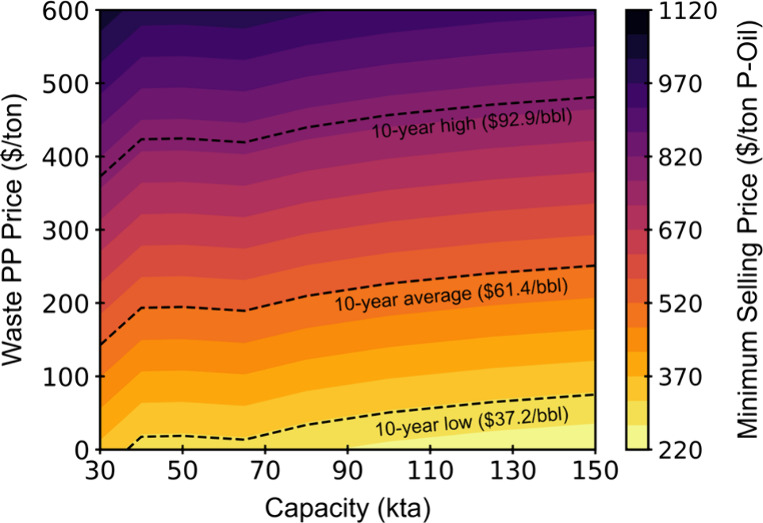
Minimum selling price of P-Oil across different plant capacities and feedstock prices, with 10-year crude oil price extremes and average marked for enhanced comparison. A transportation distance of 80 miles is assumed for facilities with capacities below 50 kta, 200 miles for capacities above 50 kta, and 140 miles for the 50 kta case.

A noticeable trend in this analysis is observed around the 50 kta threshold where the transition from modular to centralized facility configuration occurs (Figure 5). This indicates that a modular plant at the larger end of the capacity spectrum can possibly have better economics compared to a centralized facility at the smaller end. This behavior arises from the expansion of the feedstock supply radius, whereby increased transportation costs can partially or fully offset the economic benefits associated with economies of scale. Furthermore, our analysis shows that scaling beyond 90 kta yields marginal to potentially no additional economic improvement. These findings contrast in part with most studies which showed that scaling up often enhances profitability,^22,23,31,63–65^ and instead reflect current trend toward decentralized designs with lower processing capacities which eliminates or significantly reduce transportation cost. Our economic model further supports this trend: a 40 kta plant colocated with an existing MRF (i.e., assuming negligible feedstock transportation distance) results in an MSP of $418/ton under 2023 average waste PP price, approximately 1.2% lower than the baseline 100 kta case. This highlights the economic potential of smaller, decentralized facilities while emphasizing the critical role and complexity of logistics in overall system performance.

While alternative transportation modes such as rail or waterways can offer lower unit transportation costs compared to trucking, their evaluation would be most meaningful within the context of a comprehensive supply chain analysis that includes feedstock logistics and plant location optimization. Additionally, reliance on rail or water transport would significantly constrain feasible plant locations due to the limited accessibility of such infrastructure, particularly for the distributed and modular low-capacity systems considered in this study. In practice, separated waste PP would still require initial and final transport by trucks (e.g., from MRFs to rail terminals or ports, and from these points to the pyrolysis facility), adding logistical complexity and additional handling steps. Moreover, the comparatively limited network coverage of railways and waterways may lead to increased overall transport distances.

Nevertheless, while the analysis based on 2023 feedstock prices indicates economic feasibility across all examined capacities, increasing demand is expected to drive feedstock prices toward levels comparable to waste HDPE. Under such conditions, the resulting MSP exceeds the peak crude oil prices observed over the past decade,^58^ regardless of processing capacity (see Table S10 in the SI for a comprehensive comparison across capacities and feedstock prices).

### 3.4 Probabilistic Economic Feasibility

A probabilistic economic feasibility analysis was conducted (at baseline capacity and under reaction condition yielding lowest MSP) to evaluate the likelihood of achieving target economic performance under uncertainty in key parameters, thereby supporintg more informed decision making. The MSP exceeds the average price of crude oil in 2023 ($74.6/bbl or equivalently $634/ton) in more than 96% of simulated scenarios, with fewer than 4% simulated scenarios led to an MSP below this benchmark. The simulation results spanned a wide range, with MSPs varying from a minimum of $354/ton to a maximum of $2495/ton. The mean value across all 27000 trials was $1137/ton, significantly above the crude oil benchmark. The 5th, 25th, 75th, and 95th percentiles were $652, $848, $1342, and $1866 per ton, respectively, representing both the interquartile range (the central 50% of data) and a 90% confidence interval for the MSPs.

While comparing the MSPs to the average crude oil price in 2023 provides a perspective on potential profitability of the process, this approach does not account for the historical fluctuations in crude oil prices. Therefore, MSPs obtained from this analysis were also compared against the monthly crude oil prices in the US from 2014 to 2023. Element-wise comparison shows that MSPs exceed historical monthly crude oil prices in over 95% of the scenarios, indicating that they consistently surpassed crude oil market prices over the observed period (Figure 6A). This probabilistic approach highlights the uncertainties and challenges associated with achieving cost competitiveness for pyrolysis oil, indicating that such systems are unlikely to be economically viable in the absence of additional credits or incentives.

**Figure 6:**
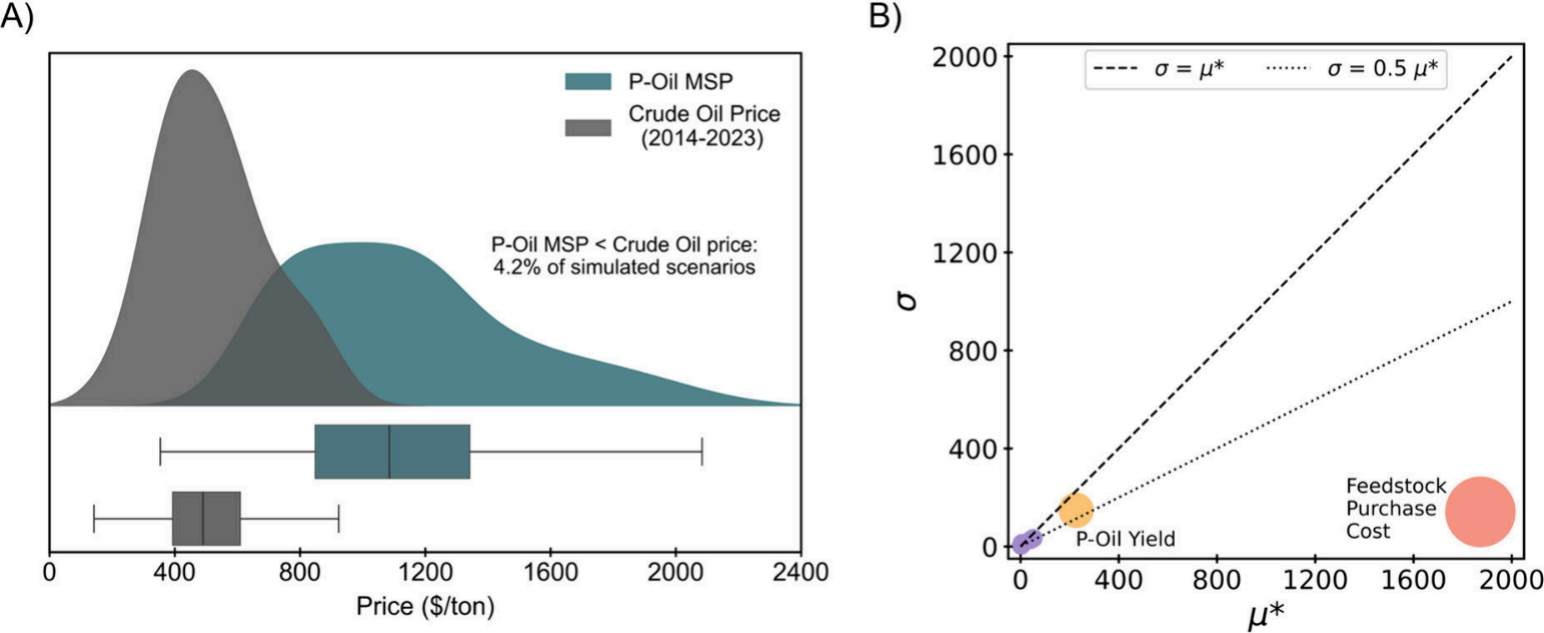
Results of the Monte Carlo uncertainty analysis. (A) Kernel density estimation plots showing the distribution of the minimum selling price of P-Oil for a 100 kta facility and the 10-year price distribution of crude oil. A bandwidth adjustment was applied to the kernel density estimations for improved visualization. (B) Morris global sensitivity analysis results showing μ* and σ indices, representing the overall influence and the degree of interaction and nonlinearity of each input parameter on minimum selling price, respectively.

To further examine the factors driving the observed MSP range, a Morris global sensitivity analysis was conducted to quantify the relative influence of each input parameter on MSP. The results (Figure 6B) identify two key parameters. First, feedstock purchase cost which emerge as the dominant factor, exhibiting a strong and nearly linear influence on MSP, as indicated by a large μ* and a low σ (σ/μ* ≪ 0.5). Second, P-Oil yield, with an impact that is moderate (μ* lower than feedstock cost but higher than other parameters), highly nonlinear (σ/μ* > 0.5), and monotonic (σ/μ* < 1). All remaining parameters exhibit comparatively minor contributions (Table S13 in the SI).

It should be noted that the strong influence of feedstock purchase cost is partly due to its wider variation range relative to other parameters. Nonetheless, this range is grounded in historical fluctuations in polyolefin prices^47^ and accounts for potential demand-driven increases associated with the deployment of this recycling technology. For P-Oil yield, the values used in this study were derived from a laboratory-scale kinetic model^46^ that already predicts high polypropylene conversion and high selectivity toward liquid products. Consequently, the assumed yields are near the upper end of the practically achievable range. Substantially higher yields in alternative pyrolysis configurations may therefore be difficult to realize, particularly at industrial scales where heat and mass transfer limitations can negatively affect system performance.

The observed sensitivity trends are also consistent with the mathematical structure of the MSP calculation. The MSP was determined by solving for a net present value (NPV) of zero:

$$\mathrm{NPV}=0=\underset{i}{\sum }\frac{\mathrm{net cash flow}}{{\left(1+r\right)}^{i}}$$

which can be simplified conceptually as

$$\mathrm{MSP}=a\times \frac{\mathrm{cost}}{m_{P\_Oil}}$$

where “cost” represents the total production costs, *m*_P_Oil_ represents the amount of P-Oil produced, and *a* is a constant term associated with factors such as discount rate, plant lifetime, and other economic assumptions. This mathematical formulation shows that MSP increases directly with production cost while varying inversely with produced amount of P-Oil, which is consistent with the trends identified in the Morris sensitivity analysis.

Overall, although the uncertainty analysis is specific to the assumptions adopted in this study, the global sensitivity analysis suggests that feedstock cost is likely to remain a critical determinant of economic performance across different process configurations. P-Oil yield also remains an important parameter; however, because the baseline assumptions already correspond to relatively high yields, only limited additional improvements may be achievable in alternative configurations or with different catalysts. Consequently, similar cost-competitiveness challenges may persist across alternative pyrolysis systems, although the magnitude of their impact may vary depending on process design and techno-economic assumptions.

## 4 Conclusions

This study examines the economic viability of a recycling facility that processes polypropylene waste, supplied as bales from MRFs, into pyrolysis oil. The produced oil serves as a substitute for crude oil in conventional refineries. The baseline capacity of the facility is set at 100 kta of waste polypropylene per year. This study shows that selecting proper operating conditions can reduce the minimum selling price of pyrolysis oil by up to 20%. Using 2023 average prices, MSP of pyrolysis oil is estimated to be $420/ton, 33% lower than the average US crude oil price for that year. This price advantage persists as long as the feedstock cost remains below $320/ton. Despite this, we firmly believe that an increase in purchase cost of feedstock is inevitable as operation of the facility would significantly increase the demand for waste polypropylene in the region. If the cost of waste polypropylene were to equal that of waste HDPE, the minimum selling price of pyrolysis oil would soar to $1480/ton, which is more than 2 times higher than crude oil price intended to replace. Sensitivity analysis revealed that the feedstock purchase cost, capital investments, and feedstock transportation are the most influential factors on profitability of the process.

Our scenario analyses indicate that reducing product storage duration from 28 to 14 days can decrease the minimum selling price by 7%. However, this reduction may come at the cost of reduced operational flexibility and an increased likelihood of shutdowns, which could negatively impact profitability. Each additional shutdown beyond the planned 35 days can, on average, increase the minimum selling price by $0.51/ton. The study also demonstrates that scaling beyond 90 kta offers limited economic benefits. Even at the highest capacity examined in this study (150 kta), the minimum selling price remains significantly higher than peak crude oil price over the past decade if feedstock costs match those of waste HDPE. Uncertainty analysis shows that the minimum selling price exceeds the historical crude oil prices in more than 95% of simulated scenarios. This variability reflects the high financial risk associated with investment and underscores the limited economic viability of pyrolysis-based recycling at scale under current and projected market conditions. While promising from a circular economy perspective, the technology’s long-term profitability is unlikely without targeted policy interventions. These may include financial incentives or regulatory measures that account for the environmental costs of fossil fuel use and promote circularity. This conclusion aligns with prior studies that emphasize the critical role of policy support in enabling the long-term commercial feasibility of pyrolysis technologies.^27,32,63^

This analysis provides a foundational framework for the economic viability of the process; however, the transition from experimental microreactor data to a commercial scale introduces inherent complexities in heat transfer, mass transfer, and operational yields. While these factors are partially addressed through the uncertainty analysis in this study, it is important to note that such models are intrinsically sensitive to the underlying assumptions and distribution ranges selected. Nevertheless, even when based on laboratory-scale data, techno-economic analysis serves as a structured decision-support tool that identifies economic bottlenecks, quantifies key cost drivers, and defines the performance targets required for commercial feasibility.

The integration of a kinetic model in place of static yield data represents a significant improvement over conventional system analysis studies; nonetheless, the present implementation is still constrained by the simplicity and limitations of the kinetic model we employed in this study. Future research should prioritize the adoption of more advanced kinetic models capable of tracking the formation of a wider range of chemical species and accounting for the influence of additional operating variables, such as pressure, catalyst type, and other factors. Such enhanced models would allow for a more comprehensive evaluation of how operating/reaction conditions impact process sustainability and enable more sophisticated sensitivity and scenario analyses.

## Supplementary Material



## Notes

Section S1, Aspen Plus process flow diagrams and associated mass and energy balance tables; Section S2, assumptions and methodological details; Section S3, supplementary economic calculation data; Section S4, cash-flow tables and scenario analysis results; Section S5, uncertainty analysis methodology and results; Section S6, analysis of operational downtime impacts; Section S7, comparison of results with literature data (PDF)
