# Supplementary material for: A Kinetic Model-Driven Techno-Economic Analysis of Plastic Pyrolysis: Linking Process Dynamics to Economic Viability
Source: Ind Eng Chem Res. 2026 May 27;65(22):11631–41. doi: 10.1021/acs.iecr.6c00693 (PMC13262053; doi:10.1021/acs.iecr.6c00693)
Supplement: Supplementary file 1 [file ie6c00693_si_002.pdf]

Supplementary information

A Kinetic Model-Driven Techno-Economic Analysis of Plastic Pyrolysis: Linking Process Dynamics to Economic Viability

Farhad Zaker Hosseiny[1], Rui Shi[1,2,*]

[1] Department of Chemical Engineering, Pennsylvania State University, University Park, PA 16802, USA.

[2] Institute of Energy and the Environment, The Pennsylvania State University, University Park, PA 16802, USA

* Corresponding author E-mail: rms6987@psu.edu

Number of Pages: 18

Number of Tables: 13

Number of Figures: 6

Supplemental Items

Sections:

1- Process simulation 1
2- Economic assumptions 3
3- Process economics estimation results 4
4- Cash-flow tables and scenario analyses results 7
5- Uncertainty analysis 10
6- Impact of operational downtimes 13
7- Results comparisons with literature data 14
8- References 15

Supporting Tables:

Table S1: Stream Summary from ASPEN process simulation 1
Table S2: Utility summary 2
Table S3: Arrhenius parameters of the kinetic model used in this work 2
Table S4: Underlying assumptions in economic evaluations 3
Table S5: Equipment cost summary 4
Table S6: Fixed capital investment from APEA 5
Table S7: Capital investment and operational cost breakdown 6
Table S8: Discounted cash-flow table 7
Table S9: Summary of scenario analyses for various capacities and storage durations 8
Table S10: Minimum selling price of pyrolysis oil at various capacities and feedstock prices 9
Table S11: Techno-economic parameters subject to uncertainty analysis 10
Table S12: K-S test results for distribution fitting 12
Table S13: Morris global sensitivity analysis results 12

Supporting Figures:

Figure S1: Process flow diagram in process simulator 1
Figure S2: Breakdown of area-specific costs within the pyrolysis facility 6
Figure S3: Variation in cooling and compressor duties with oil yield 11
Figure S4: Price fluctuation of waste PP and HDPE 11
Figure S5: Impact of process downtime on profitability. 13
Figure S6: Minimum selling price and break-even feedstock price comparisons with literature. 14

1- Process simulation

This section provides detailed information on the process simulation in Aspen Plus, including the actual flowsheet, mass and energy balance results for the baseline capacity of 100 kta, and the reaction rate parameters used in this study. The flowsheet remained the same across all other processing capacities considered.

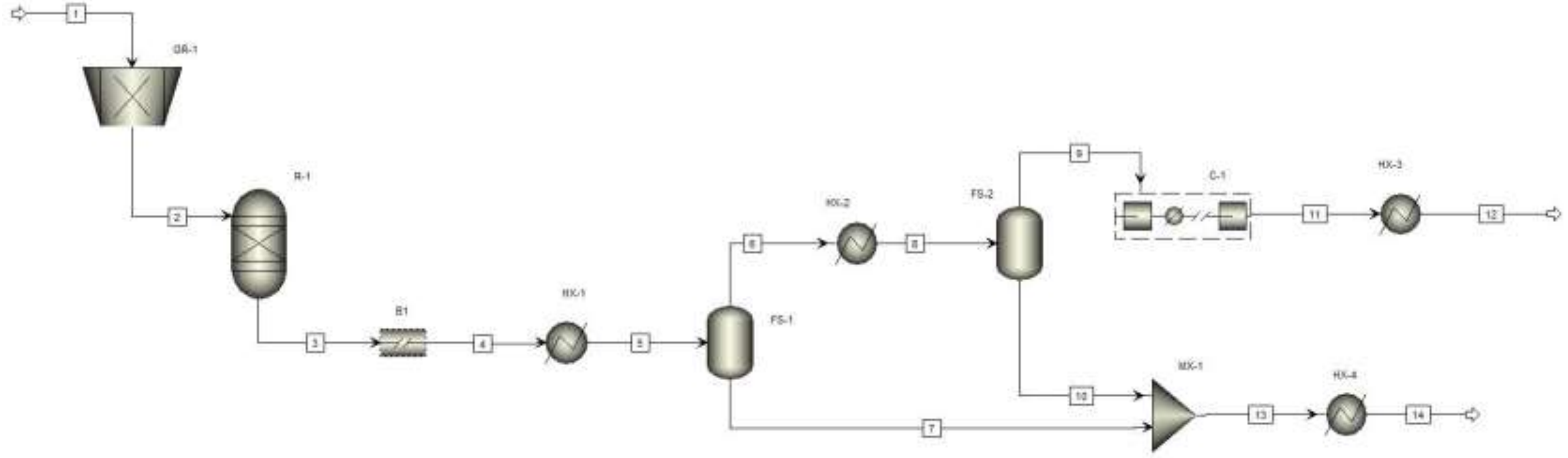

Figure S1: Process flow diagram in process simulator

Table S1: Stream Summary from ASPEN process simulation (baseline capacity of 100 kta, Reaction condition: T=525 °C, VRT=6 s)

	Units	1	2	3	4	5	6	7	8	9	10	11	12	13	14
Temperature	C	25	25	525	525	100	100	100	30	30	30	100	30	87.2	30
Pressure	bar	1	1	1	1	1	1	1	1	1	1	13	13	1	1
Total Mass Flow	kg/hr	12626.3	12626.3	12626.3	12626.3	12626.3	3715.9	8910.3	3715.9	1791.5	1924.4	1791.5	1791.5	10834.8	10834.8
Gas Mass Flow	kg/hr	0.0	0.0	1766.8	1766.8	1766.8	1711.6	55.1	1711.6	1644.6	67.0	1644.6	1644.6	122.2	122.2
Light Oil Mass Flow	kg/hr	0.0	0.0	5507.4	5507.4	5507.4	1903.6	3603.9	1903.6	145.9	1757.7	145.9	145.9	5361.5	5361.5
Heavy Oil Mass Flow	kg/hr	0.0	0.0	4786.9	4786.9	4786.9	30.7	4756.1	30.7	0.0	30.7	0.0	0.0	4786.8	4786.8
Wax Mass Flow	kg/hr	0.0	0.0	16.1	16.1	16.1	1.8E-05	16.1	1.8E-05	1.6E-14	1.8E-05	1.6E-14	1.6E-14	16.1	16.1
Aromatics Mass Flow	kg/hr	0.0	0.0	549.1	549.1	549.1	70.0	479.2	70.0	1.0	69.0	1.0	1.0	548.2	548.2
PP Mass Flow	kg/hr	12626.3	12626.3	0.0	0.0	0.0	0.0	0.0	0.0	0.0	0.0	0.0	0.0	0.0	0.0
Mass Vapor Fraction	-	0	0	1	1	0.295	1	0	0.48	1	0	1	0	0.005	0
Mass Liquid Fraction	-	0	0	0	0	0.705	0	1	0.52	0	1	0	1	0.995	1
Mass Solid Fraction	-	1	1	0	0	0	0	0	0	0	0	0	0	0	0

Table S2: Utility summary (baseline capacity of 100 kta, Reaction condition: T=525 °C, VRT=6 s)

Equipment ID - type	Duty (GJ/h)
Cooling water	
HX-1 – Heat exchanger	16.84
HX-2 – Heat exchanger	1.11
HX-3 – Heat exchanger	0.82
HX-4 – Heat exchanger	1.34
C-1 – Compressor intercooler	0.22
Electricity	
Gr-1 – Grinder	0.41
C-1 – Multistage compressor	4.99
Natural gas	
R-1 – Pyrolysis reactor	19.95

Table S3: Arrhenius parameters of the kinetic model used in this work

Reaction constant	A (1/s)	E_a (kJ/mol)
k_1 (polymer → wax)	1.26×10^{13}	2.01×10^{2}
k_2 (polymer → heavy oiil)	2.44×10^{3}	3.88×10^{1}
k_3 (polymer → light oil)	1.28×10^{2}	2.09×10^{1}
k_4 (polymer → gas)	9.94×10^{7}	1.15×10^{2}
k_5 (wax → heavy oil)	5.29×10^{-1}	7.22×10^{0}
k_6 (wax → gas)	2.31×10^{10}	2.77×10^{2}
k_7 (light oil → gas)	1.36×10^{-3}	9.18×10^{-1}
k_8 (heavy oil → light oil)	1.14×10^{8}	1.47×10^{2}
k_9 (wax → light oil)	1.89×10^{9}	1.47×10^{2}
k_{10} (gas → aromatics)	4.60×10^{-2}	4.14×10^{-3}

The original kinetic model data [1] was modified by reducing all pre-exponential factors (Ai values) by a factor of 10 and by switching the Ai and Ea parameters between the k3 and k4 reaction constants.[1] These modifications were necessary to replicate predictions of the kinetic model presented in the original article, whose kinetic data remains unrevised as of the time of publishing this manuscript.

[1] Based on communication with article author

2- Economic assumptions

The assumptions underlying the economic calculations, including key cost parameters, utility costs, raw material prices, and operational conditions, are summarized in Table S4. The waste PP transportation distance is assumed to be 80 miles for facilities with capacities below 50 kta and 200 miles for those above 50 kta, based on an industry survey [2]. For the 50 kta case, an intermediate value of 140 miles is used, as the survey does not explicitly classify this capacity as either modular or centralized.

Service cost parameters, such as cooling water and hauling prices, were adjusted to reflect 2023 values using relevant Producer Price Indices (PPI) by Industry from the US Federal Reserve Economic Data (FRED) [3,4] . Specifically, the Producer Price Index by Industry: Utilities (PCU221221) was used to adjust the cooling water price, and the Producer Price Index by Industry: Truck Transportation (PCU484484) was applied to update the waste hauling price. These indices account for inflation and sector-specific cost variations over time.

Table S4: Underlying assumptions in economic evaluations (prices represents 2023 averages)

Parameter	Value	Unit	Reference(s)
Waste polypropylene price	143	$/ton	[5]
Industrial electricity price	8.04	Cents/kWh	[6]
Cooling water price[1]	0.27	$/GJ	[7]
Natural gas price	0.2	$/kg	[8,9]
Pyrolysis gas sale price[2]	0.192	$/kg	
Waste hauling price[1]	0.47	$/(ton.mile)	[10]
Average hauling distance estimate	80, 140, 200	mile	[2]
Natural gas lower heating value	47.14	MJ/kg	[11]
Pyrolysis gas lower heating value	45.8	MJ/kg	[12]
Furnace heating efficiency	70	%	[13,14]
Grinder power input	110	kWh/ton	[15,16]
Working to fixed capital investment ratio	10	%	
Discount rate	15	%	
Tax rate	26	%	
Depreciation method	7-year MACRS	-	
Plant life	30 (3+27)	year	
On-stream time	7920 (330x24)	hour/year	

[1] Updated by price index

[2] Calculated based on natural gas price and LHV ratios

3- Process economics estimation results

Aspen Process Economic Analyzer (APEA) was used to calculate equipment costs, capital investments, and fixed operational costs (in 2019 dollars). The fixed operational costs include supervision and operating labor costs, maintenance costs, operating charges, plant overhead costs, and general and administrative (G&A) costs. The Chemical Engineering Plant Cost Index (CEPCI), published by Chemical Engineering magazine [17] was used to update the total capital investment to 2023 dollars. Labor costs were adjusted using the Employment Cost Index: Wages and Salaries: Private Industry Workers (ECIWAG), provided by the US Federal Reserve Economic Data [18]. Maintenance costs were updated using Producer Price Index by Commodity: Repair and Maintenance Services (Partial): Commercial and Industrial Machinery and Equipment Repair and Maintenance (WPU551) [19]. Figure S2 provides an overview of the costs associated with each section. This analysis considers only utility and equipment costs, as other components of capital investments and operational costs apply to the plant as a whole. In this analysis, the annuity method [13] with a 10% interest rate is used to annualize the equipment cost. The analysis reveals that the storage area incurs the highest area-specific cost to the plant, closely followed by the pyrolysis area.

Table S5: Equipment cost summary[1] (baseline capacity of 100 kta)

Equipment - Description	Tag No.	Equipment Cost ($)	Total Installed Cost ($)
Furnaces (x2) - Pyrolysis unit	R-1	1,693,444	2,116,807
Cooling Tow ers (x2) - Packaged cooling tow er, factory assembly	CT-1	161,866	469,743
Heat Exchangers - Fixed tube, float. head, u-tube exchanger	HX-1	20,762	128,729
Heat Exchangers - Fixed tube, float. head, u-tube exchanger	HX-2	22,162	98,630
Heat Exchangers - Fixed tube, float. head, u-tube exchanger	HX-3	18,862	84,100
Heat Exchangers - Fixed tube, float. head, u-tube exchanger	HX-4	16,762	78,702
Crushers - Nonreversible hammermill, med hard material	GR-1	199,460	208,684
Conveyors - Belt conveyor	CONV-1	71,444	100,636
Conveyors (x2) - Belt conveyor	CONV-2	95,735	138,492
Flash Drum - Vertical process vessel	FS-1	25,971	140,261
Flash Drum - Vertical process vessel	FS-2	25,971	132,289
Gas Compressors - Multistage centrifugal compressor	C-1	1,064,203	1,209,404
P-Oil Storage Tanks (x2) - Flat bottom storage tank, floating roof	STG-1[2]	1,986,200	3,151,986
Liquified P-Gas Storage Tanks (x3) - Vertical process vessel	STG-2[2]	5,355,855	6,381,244

[1] Price show n are not updated to 2023 dollars

[2] Not show n in Figure S1

Table S6: Fixed capital investment from APEA (baseline capacity of 100 kta)

Account	Labor Cost ($)	Matl Cost ($)	Total Cost ($)	Percentages (%)
Equipment	261,796	10,885,900	11,147,696	61.0% of TDC
Piping	313,915	724,571	1,038,486	5.7% of TDC
Civil	784,614	1,427,154	2,211,768	12.1% of TDC
Steel	42,482	235,769	278,251	1.5% of TDC
Instruments	191,595	1,001,099	1,192,694	6.5% of TDC
Electrical	262,142	1,861,038	2,123,180	11.6% of TDC
Insulation	40,518	38,500	79,018	0.4% of TDC
Paint	146,461	70,672	217,133	1.2% of TDC
Total Direct Field Costs	2,043,523 TDL	16,244,703 TDM	18,288,226 TDC	100.0% of TDC
Indirect Field Costs			2,734,000 IFC	133.8% of TDL
Total Field Costs			21,022,226 TFC	66.5% of TIC
Freight			649,800	4.0% of TDM
Taxes and Permits			1,015,300	5.6% of TDC
Engineering and HO			2,389,400	7.6% of TIC
Other Project Costs			1,726,807	5.5% of TIC
Contingency			4,824,636	15.3% of TIC
Total Non-Field Costs			10,605,943	33.5% of TIC
Project Total Costs			31,628,169 TIC	172.9% of TDC

Table S7: Capital investment and operational cost breakdown (baseline capacity of 100 kta)

Plant capital investments ($)	
Fixed capital investment	41,540,932
Working capital	4,154,093
Total depreciable capital	29,682,534
Total capital investment	45,695,026
**Plant operational cost ($/year)**	
Raw material	14,300,000
Feedstock transportation	9,446,849
Utilities	1,958,831
Supervision and operating labor cost	1,084,047
Maintenance cost	470,234
Operating charges	230,000
Plant overhead costs	648,000
General & Administrative costs	1,544,574

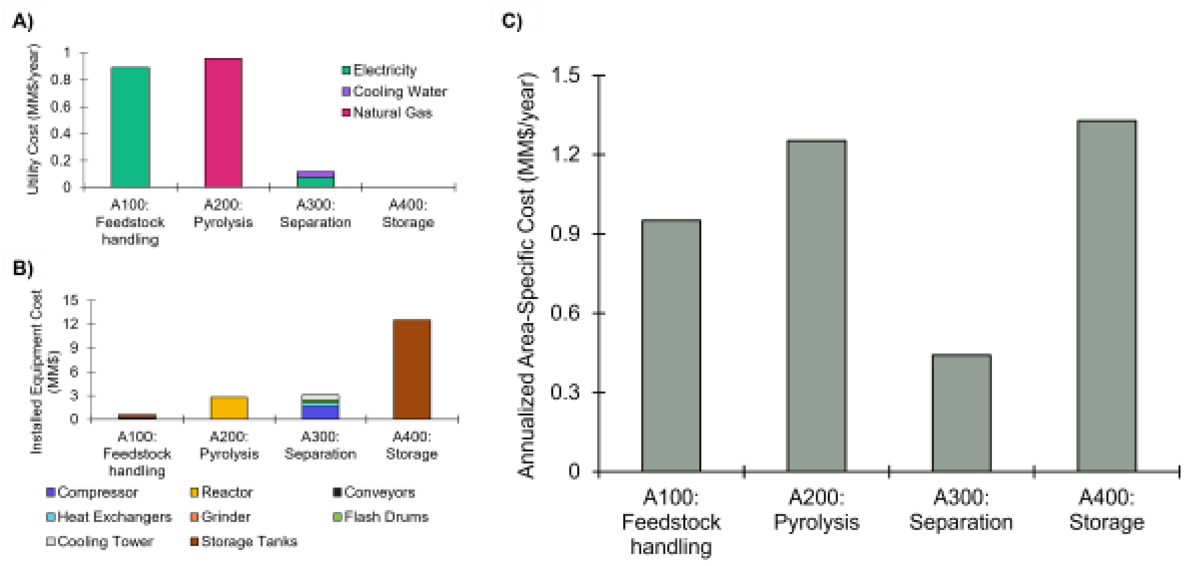

Figure S2: Breakdown of area-specific costs within the pyrolysis facility: A) utility costs, B) installed equipment cost, and C) total annualized cost, obtained by combining utility costs with annualized equipment costs. Equipment costs are annualized with an interest rate of 10% using annuity method. All results correspond to a plant capacity of 100 kta.

4- Cash-flow tables and scenario analyses results

Table S8 presents the discounted cashflow table. Capital costs during the construction period are allocated as follows: 30% of the fixed capital investment in year 1, 50% in year 2, and 20% of the fixed capital investment along with working capital in year 3. In the first year of operation (year 4), sales revenue and variable operating costs are assumed to be halved to account for losses and downtime during the initial plant start-up. The discount factor (10%) is assumed to be 1 throughout the construction period and is applied starting from year 4. Depreciation is calculated using the 7-year MACRS (Modified Accelerated Cost Recovery System) method.

Table S8: Discounted cash-flow table (baseline capacity of 100 kta, Reaction condition: T=525 ^oC and VRT=6 s, MSP=$423.6/ton, NPV=0)

Years	Sales Revenue	Costs	Gross Profit	Depreciation	Taxable income	Tax Paid	Cash Flow	discount factor	Present value of cash flow
1	0	12,462,280	-12,462,280	0	-12,462,280	0	-12,462,280	1.000	-12,462,280
2	0	20,770,466	-20,770,466	0	-20,770,466	0	-20,770,466	1.000	-20,770,466
3	0	12,462,280	-12,462,280	0	-12,462,280	0	-12,462,280	1.000	-12,462,280
4	19,536,135	16,829,695	2,706,441	4,509,961	-1,803,521	0	2,706,441	0.870	2,353,427
5	39,072,270	29,682,534	9,389,736	7,729,108	1,660,628	431,763	8,957,973	0.756	6,773,514
6	39,072,270	29,682,534	9,389,736	5,519,890	3,869,846	1,006,160	8,383,576	0.658	5,512,337
7	39,072,270	29,682,534	9,389,736	3,941,877	5,447,859	1,416,443	7,973,292	0.572	4,558,756
8	39,072,270	29,682,534	9,389,736	2,818,331	6,571,404	1,708,565	7,681,171	0.497	3,818,899
9	39,072,270	29,682,534	9,389,736	2,815,175	6,574,561	1,709,386	7,680,350	0.432	3,320,427
10	39,072,270	29,682,534	9,389,736	2,818,331	6,571,404	1,708,565	7,681,171	0.376	2,887,637
11	39,072,270	29,682,534	9,389,736	1,407,588	7,982,148	2,075,359	7,314,377	0.327	2,391,083
12	39,072,270	29,682,534	9,389,736	0	9,389,736	2,441,331	6,948,404	0.284	1,975,170
13	39,072,270	29,682,534	9,389,736	0	9,389,736	2,441,331	6,948,404	0.247	1,717,539
14	39,072,270	29,682,534	9,389,736	0	9,389,736	2,441,331	6,948,404	0.215	1,493,512
15	39,072,270	29,682,534	9,389,736	0	9,389,736	2,441,331	6,948,404	0.187	1,298,706
16	39,072,270	29,682,534	9,389,736	0	9,389,736	2,441,331	6,948,404	0.163	1,129,310
17	39,072,270	29,682,534	9,389,736	0	9,389,736	2,441,331	6,948,404	0.141	982,009
18	39,072,270	29,682,534	9,389,736	0	9,389,736	2,441,331	6,948,404	0.123	853,921
19	39,072,270	29,682,534	9,389,736	0	9,389,736	2,441,331	6,948,404	0.107	742,540
20	39,072,270	29,682,534	9,389,736	0	9,389,736	2,441,331	6,948,404	0.093	645,687
21	39,072,270	29,682,534	9,389,736	0	9,389,736	2,441,331	6,948,404	0.081	561,467
22	39,072,270	29,682,534	9,389,736	0	9,389,736	2,441,331	6,948,404	0.070	488,232
23	39,072,270	29,682,534	9,389,736	0	9,389,736	2,441,331	6,948,404	0.061	424,549
24	39,072,270	29,682,534	9,389,736	0	9,389,736	2,441,331	6,948,404	0.053	369,173
25	39,072,270	29,682,534	9,389,736	0	9,389,736	2,441,331	6,948,404	0.046	321,020
26	39,072,270	29,682,534	9,389,736	0	9,389,736	2,441,331	6,948,404	0.040	279,148
27	39,072,270	29,682,534	9,389,736	0	9,389,736	2,441,331	6,948,404	0.035	242,738
28	39,072,270	29,682,534	9,389,736	0	9,389,736	2,441,331	6,948,404	0.030	211,076
29	39,072,270	29,682,534	9,389,736	0	9,389,736	2,441,331	6,948,404	0.026	183,544
30	39,072,270	29,682,534	9,389,736	0	9,389,736	2,441,331	6,948,404	0.023	159,604

Table S9: Summary of scenario analyses for various capacities and storage durations, based on the assumptions outlined in Table S4

Scenario	30 kta	40 kta	50 kta	65 kta	80 kta	100 kta	125 kta	150 kta	100 kta 21-day storage	100 kta 14-day storage
Total Capital Investment ($)	27,013,316	29,589,981	32,379,596	37,758,892	41,012,920	45,695,026	51,123,015	55,943,192	40,620,361	34,641,694
Annual operational Cost ($/year)	8,640,736	10,827,159	14,437,931	20,038,385	24,175,571	29,682,536	36,573,448	43,458,770	29,575,910	29,424,390
Annual Income ($/year)	17,138,599	22,851,465	28,564,331	37,133,631	45,702,930	57,128,663	71,410,829	85,692,994	57,128,663	57,128,663
Annual Gross Earnings ($/year)	8,497,862	12,024,306	14,126,400	17,095,246	21,527,359	27,446,127	34,837,380	42,234,224	27,552,753	27,704,273
Break-Even Feedstock Price ($/ton)	239.4	285.2	291.7	286.4	306.6	323.6	337.7	348.0	334.7	348.0
Minimum Selling Price ($/ton)	521.6	462.2	460.7	521.6	443.3	423.6	407.1	395.1	410.6	395.1
NPV ($)	13,247,966	26,775,876	33,721,054	42,246,847	59,241,234	81,630,388	109,963,972	138,868,994	86,619,077	92,593,790
IRR (%)	22.38	28.17	30.03	31.10	35.40	39.80	44.39	48.47	44.09	50.60

The minimum selling price serves as the primary economic indicator in this study. The net present value (NPV) and internal rate of return (IRR) were calculated for informational purposes only, assuming the selling price of pyrolysis oil matches that of crude oil, in addition to the assumptions detailed in Table S4.

Table S10: Minimum selling price of pyrolysis oil at various capacities and feedstock prices

Feedstock price ($/ton)	30 kta	40 kta	50 kta	65 kta	80 kta	100 kta	125 kta	150 kta
0	355.0	295.6	294.1	300.3	276.7	256.9	240.4	228.4
50	413.2	353.9	352.4	358.5	335.0	315.2	298.7	286.7
100	471.5	412.1	410.6	416.8	393.2	373.5	357.0	345.0
109	482.0	422.6	421.1	427.3	403.7	384.0	367.4	355.4
137	514.6	455.2	453.7	459.9	436.3	416.6	400.1	388.1
171	554.3	494.9	493.4	499.5	476.0	456.2	439.7	427.7
200	588.0	528.7	527.2	533.3	509.8	490.0	473.5	461.5
215	605.5	546.1	544.6	550.8	527.2	507.5	491.0	479.0
270	669.6	610.2	608.7	614.9	591.3	571.6	555.1	543.1
300	704.6	645.2	643.7	649.9	626.3	606.5	590.0	578.0
338	748.9	689.5	688.0	694.2	670.6	650.8	634.3	622.3
400	821.1	761.7	760.2	766.4	742.8	723.1	706.6	694.6
424	849.1	789.7	788.2	794.4	770.8	751.0	734.5	722.5
500	937.7	878.3	876.8	882.9	859.4	839.6	823.1	811.1
532	974.9	915.6	914.1	920.2	896.7	876.9	860.4	848.4
600	1054.2	994.8	993.3	999.5	975.9	956.1	939.6	927.6
650	1112.5	1053.1	1051.6	1057.7	1034.2	1014.4	997.9	985.9
667	1132.3	1072.9	1071.4	1077.6	1054.0	1034.2	1017.7	1005.7
700	1170.7	1111.3	1109.8	1116.0	1092.4	1072.7	1056.2	1044.2
800	1287.3	1227.9	1226.4	1232.5	1209.0	1189.2	1172.7	1160.7
837	1330.4	1271.0	1269.5	1275.7	1252.1	1232.3	1215.8	1203.8
1050	1578.6	1519.2	1517.7	1523.9	1500.3	1480.5	1464.0	1452.0

5- Uncertainty analysis

In this analysis, uniform distribution was applied to highly uncertain parameters, such as "Required Energy for Pyrolysis" and "Transportation Distance", as well as to parameters that influence outcomes and have a degree of arbitrariness, such as "Discount Rate" and "Tax Rate". For the process parameters, a triangular distribution was chosen. This decision was based on the high reliability of parameters obtained from Aspen, making it reasonable to assume a triangular distribution with the mode equal to the baseline value and a ±15% to ±20% deviation serving as the minimum and maximum values. Additionally, the limited number of data points (except for "Feedstock purchase cost") made it impractical to assume or fit other probability distributions, such as the normal distribution. For "Natural gas price" and "Electricity price", the minimum, maximum, and mode values were based on the respective minimum, maximum, and average prices in the US in 2023 [6]. Additionally, correlations between (1) cooling water usage and P-Oil yield and (2) compressor power requirements and P-Oil yield were incorporated in the uncertainty analysis to better capture the dynamic behavior of the process, as illustrated in Figure S3.

Table S11: Techno-economic parameters subject to uncertainty analysis

Parameter	Baseline Value	Minimum Value	Maximum Value	Mode	Distribution	References for range
Fixed Capital investment ($)	31,628,169	25,302,536	37,953,803	31,628,169	Triangular	
Working to fixed capital ratio (%)	10	5	15	10	Triangular - integer	
Feedstock purchase cost ($/kg)	0.143	0.098	1.657	0.473	Triangular	[5]
Transportation Distance (mile)	200	150	300	-	Uniform - integer	
Hauling price[1] ($/(ton.mile))	0.34	0.255	0.425	-	Uniform	
Maintenance cost ($/year)	470,234	376,187	564,281	470,234	Triangular	
Fixed operational cost ($/year)	3,506,621	2,629,965	4,383,276	3,506,621	Triangular	
Tax rate (%)	26	21	31		Uniform - integer	
Time on stream (day)	330	300	340	330	Triangular - integer	
P-Oil yield (%)	85.8	70	90	85.8	Triangular	
Discount rate (%)	15	10	15	-	Uniform - integer	
Size reduction power requirement (kWh/ton)	109.8	75	300	100	Triangular	[15,20]
Compressor duty variation from estimated value (%)	0	-15	+15	0	Triangular	
Electricity price ($/kWh)	0.0806	0.0751	0.089	0.0803	Triangular	[6]
Cooling water consumption variation from estimated value (%)	0	-15	+15	0	Triangular	
Cooling water price[1] ($/GJ)	0.212	0.159	0.265	0.212	Triangular	
Required Energy for pyrolysis (MJ/kg)	1.58	1.45	2.2	-	Uniform	[21,22]
LHV natural gas (MJ/kg)	47.14	42	53	47.141	Triangular	[23]
Natural gas price ($/kg)	0.2	0.15	0.31	0.2	Triangular	[8]
Furnace efficiency (%)	70	50	80	70	Triangular	[13,14]

[1] The values shown do not reflect adjustments based on the updated Producer Price Index; however, these adjustments are included in the analysis as separate factors.

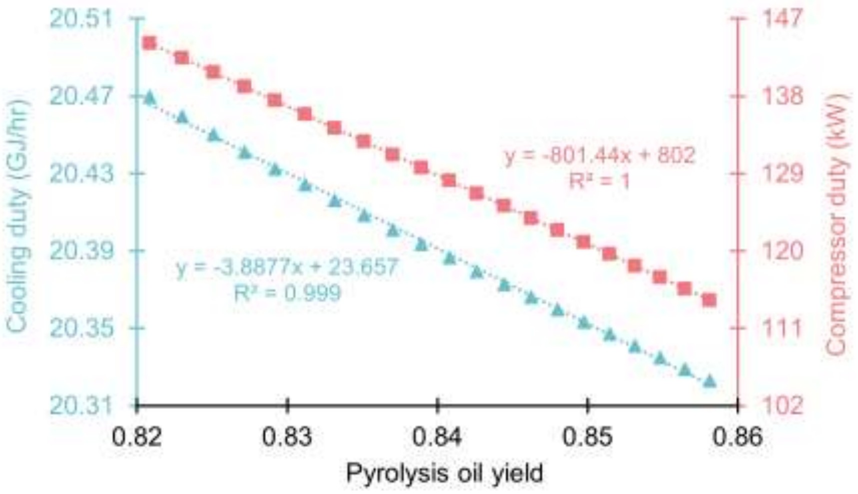

Figure S4: Variation in cooling and compressor duties with oil yield

Figure S4 illustrates the price fluctuations of polypropylene and high-density polyethylene waste bales (both natural and colored) sourced from MRFs over the past decade and in 2023 [5]. Over the decade, natural HDPE prices typically averaged around \$650/ton, colored HDPE around \$400/ton, and polypropylene around \$220/ton. However, between 2021 and 2023, there was a sharp rise and subsequent decline in prices, with the decrease reflected in the fluctuations observed in 2023. To account for these fluctuations, we employed a triangular distribution in our analysis, setting the minimum, maximum, and mode values to the minimum, maximum, and average prices of polypropylene and HDPE (natural and colored) waste observed in 2023. The inclusion of natural HDPE, particularly, was intended to capture the impact of increased demand on pricing and enhance our understanding of the recycling facility's future economic performance. While this approach provides a reasonable approximation for the current study, it is important to acknowledge that a more in-depth analysis is necessary to accurately predict future polypropylene price trends.

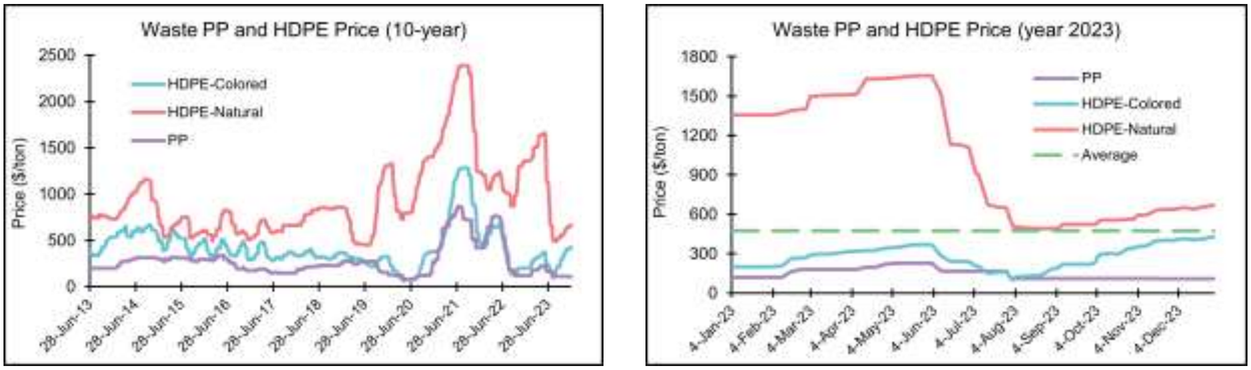

Figure S3: Price fluctuation of waste PP and HDPE between 2013 and 2023 (left) and during 2023 (right)

A minor issue with using kernel density estimates in such analyses is that they predict the probability of a minimum selling price falling outside the observed range as absolute zero, which may not be entirely accurate. Even with a larger number of samples, we cannot guarantee that the minimum and maximum values observed are global extremes. To overcome this issue, we attempted to identify the probability density function (PDF) that best fits the data. In our analysis, the "Inverse Gaussian" distribution showed the lowest K-S (Kolmogorov-Smirnov) statistic, indicating a marginally better fit to the data compared to other PDFs. However, even for this distribution, the P-value was much lower than 0.05 (Table S12), leading to the rejection of the null

hypothesis. Based on this, the data obtained from the Monte Carlo simulation does not conform to any typical probability distribution.

Table S12: K-S test results for distribution fitting

Distribution type	KS-Statistic	P-Value
Birnbaum-Saunders	0.02719	8.81E-18
Burr	0.03286	8.89E-26
Gamma	0.03203	1.62E-24
Generalized Extreme Value	0.02993	1.87E-21
Inverse Gaussian	0.02707	1.25E-17
Log-logistic	0.03388	2.28E-27
Lognormal	0.02859	1.30E-19
Nakagami	0.04169	3.14E-41

Table S13 summarizes the Morris global sensitivity analysis results for all evaluated techno-economic parameters, including the mean absolute elementary effect (μ^*) and standard deviation (σ). The μ^*values indicate the overall influence of each parameter on the MSP, while the σ values reflect the extent of nonlinear behavior and parameter interactions within the model.

Table S13: Morris global sensitivity analysis results for techno-economic parameters

**Parameter**	$\boldsymbol{\mu^*}$	$\boldsymbol{\sigma}$
Feedstock Purchase Cost	1873.30	140.31
P-Oil Yield	227.30	149.20
Transportation Distance	51.70	34.68
Unit Price for Bale Transport	41.96	27.40
Discount Rate	9.07	10.89
Fixed Capital Investment	8.86	9.84
Fixed Operational Cost	6.82	10.66
Electricity Price	6.67	10.32
Required Energy for Pyrolysis	5.82	12.49
Natural Gas LHV	4.97	7.24
Natural Gas Price	4.96	7.85
Furnace Efficiency	4.57	6.79
Maintenance Cost	4.55	7.00
Time-on-Stream	4.41	7.74
Cooling Water Price	4.03	6.63
Tax	3.13	5.12
Working Capital	2.54	4.23

6- Impact of operational downtimes

Whether caused by extended maintenance times or issues with feedstock availability and/or product shipment, production can experience downtimes that exceed what was anticipated (35-day) in our economic calculations. During downtimes, there would be no revenue stream for the system, as product sales are the sole source of income. Regarding operational costs, there would be no variable operating costs (feedstock purchase, transportation, and utility costs) during downtimes. However, fixed operational costs will remain unchanged, regardless of the operational status. In this analysis, the process downtime was varied from 25 to 85 days (35 days was the baseline in all economic calculations in this study). As shown in Figure S5, the MSP of P-Oil increases from \$419/ton to \$451/ton across the evaluated downtime range. Similarly, the break-even feedstock price decreases from \$327/ton to \$300/ton, both indicating a narrowing profitability margin. While this ~7% variation in MSP might initially appear negligible, discounted cash flow analysis using the assumed benchmark product selling price (\$634/ton) indicates a moderate impact on long-term project economics. Specifically, reducing downtime to achieve the lower MSP bound increases the NPV from approximately \$60 million to \$86 million over the assumed project lifetime and production scale. Measured against the initial capital investment of \$45.7 million, both operational scenarios maintain baseline economic viability; however, the compounding nature of continuous production at scale translates the modest MSP reduction into a moderate financial advantage. This leverage is further evidenced by the IRR, which increases from 33.7% to approximately 41%. Ultimately, the analysis demonstrates that while the direct sensitivity of MSP to downtime is slight, the compounding effect over the operational lifetime of the facility leads to a moderate difference in overall economic performance.

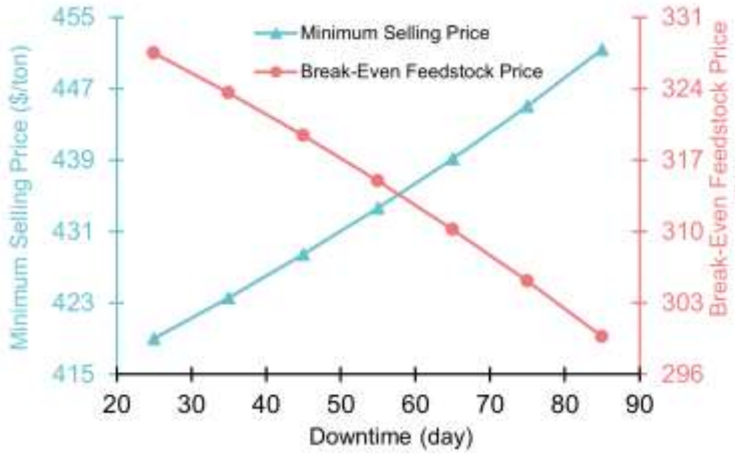

Figure S5: Impact of process downtime on profitability. The blue line and markers indicate the trend for minimum selling price, and red line and markers show the trend for break-even feedstock price. All results correspond to a plant capacity of 100 kta.

The near-linear trend in this analysis arises because variable operational costs are the primary drivers of the economic viability. Specifically, the contribution of variable operational costs to the MSP is significantly higher than that of CAPEX and fixed operational costs (306 vs. 88 vs. 62, respectively). If CAPEX or fixed operational costs had a greater influence, we would expect a more pronounced non-linear trend. While variable operational costs remain the primary driver, the combined impact of CAPEX and fixed costs is still considerable, accounting for 33% of the MSP (excluding P-Gas credits). This explains why extended downtimes continue to have a noticeable effect on the system. Our calculations reveal that each additional day of process downtime, relative to the baseline, results in an average increase of \$0.51/ton in the MSP of the product. This highlights the importance of effective maintenance and supply-chain planning to mitigate the risk of extended downtimes. If downtime can be reduced, the negative impacts on product pricing could be significantly minimized, with the 25-day downtime (MSP=\$419/ton) serving as an example of this improvement.

7- Results comparisons with literature data

Comparing the results of various TEA studies is often challenging due to differences in the underlying assumptions used in economic evaluations. These discrepancies may involve variations in discount rates for cash-flow analyses, plant lifetimes, process configurations (such as different process configurations in the separation area or inclusion of product upgrading), processing capacity, feedstock prices, product yields, and more. For this analysis, we first established correlations between plant size and both minimum selling price and break-even feedstock price. This approach allowed us to compare the TEA results of this study with those from other studies that assumed different plant capacities. As shown in Figure S6, power law equations effectively correlate minimum selling price and break-even feedstock price with plant size, achieving a high degree of accuracy ($R^2>0.99$) in both cases. It is important to note, however, that these correlations are specific to the system and economic assumptions of this study. Applying these correlations to interpolate or extrapolate results for other systems is likely to yield inaccurate or misleading outcomes. As shown in Figure S6, while our calculations for the minimum selling price and break-even feedstock price align fully or partially with some studies [24–26], they differ significantly from the greater number of other studies [27,20,16,28,29]. Upon investigating these differences, we observed that feedstock price and product yields have the most significant impact on the minimum selling price of the main product. When we adjusted these factors in our analysis to match those used in other studies, the differences in minimum selling price narrowed to within ±20%. This level of agreement appears reasonable given the variations in other economic assumptions across studies.

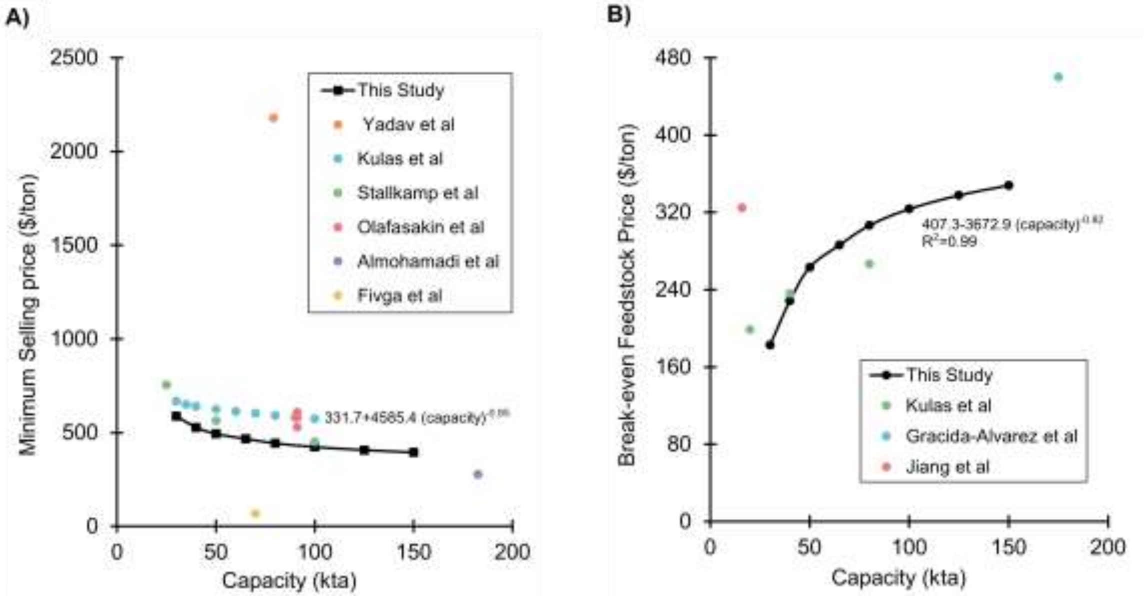

Figure S6: (A) Minimum selling price and (B) break-even feedstock price comparisons with literature data

8- References

[1] Kulas DG, Zolghadr A, Shonnard D. Micropyrolysis of Polyethylene and Polypropylene Prior to Bioconversion: The Effect of Reactor Temperature and Vapor Residence Time on Product Distribution. ACS Sustainable Chem Eng 2021;9:14443–50. https://doi.org/10.1021/acssuschemeng.1c04705.

[2] Benavides P, Gracida-Alvarez U, Lee U, Wang M. Life-cycle Analysis of Conversion of Post-Use Plastic via Pyrolysis with the GREET Model. 2022. https://doi.org/10.2172/1885570.

[3] Federal Reserve Economic Data (FRED). Producer Price Index by Industry: Utilities n.d. https://fred.stlouisfed.org/series/PCU221221 (accessed December 2, 2024).

[4] Federal Reserve Economic Data (FRED). Producer Price Index by Industry: Truck Transportation n.d. https://fred.stlouisfed.org/series/PCU484484 (accessed December 2, 2024).

[5] Recycling Markets Limited (RML). Secondary Materials Pricing n.d. https://www.recyclingmarkets.net/secondarymaterials/ (accessed May 17, 2024).

[6] U.S. Energy Information Administration (EIA). Electricity data browser n.d. https://www.eia.gov/electricity/data/browser/ (accessed December 2, 2024).

[7] Aspen Technology, Inc. Aspen Plus 2021.

[8] U.S. Energy Information Administration (EIA). United States Natural Gas Industrial Price (Dollars per Thousand Cubic Feet) n.d. https://www.eia.gov/dnav/ng/hist/n3035us3a.htm (accessed May 27, 2024).

[9] U.S. Energy Information Administration (EIA). British thermal units (Btu) - U.S. Energy Information Administration (EIA) n.d. https://www.eia.gov/energyexplained/units-and-calculators/british-thermal-units.php (accessed May 27, 2024).

[10] A Goldsmith Resources. Cost Projections For Transfer, Haul, And Disposal Of Municipal Solid Waste - Prepared for Beaufort County Council. 2014.

[11] Boundy RG, Diegel SW, Wright LL, Davis SC. Biomass Energy Data Book: Edition 4. Oak Ridge National Laboratory (ORNL); 2011. https://doi.org/10.2172/1050890.

[12] Engineering ToolBox. Fuel Gases - Heating Values n.d. https://www.engineeringtoolbox.com/heating-values-fuel-gases-d_823.html (accessed December 2, 2024).

[13] Seider WD, Lewin DR, Seader JD, Soemantri Widagdo (Chemical Engineer, R Gani, Ka Ming Ng. Product and process design principles : synthesis, analysis, and evaluation. John Wiley & Sons Inc; 2017.

[14] Garg A. Get the Most From Your Fired Heater. Chemical Engineering 2004;111:60–5.

[15] Macko M. Size Reduction by Grinding as an Important Stage in Recycling. In: Damanhuri E, editor. Post-Consumer Waste Recycling and Optimal Production, InTech; 2012. https://doi.org/10.5772/33969.

[16] Yadav G, Singh A, Dutta A, Uekert T, DesVeaux JS, Nicholson SR, et al. Techno-economic analysis and life cycle assessment for catalytic fast pyrolysis of mixed plastic waste. Energy Environ Sci 2023;16:3638–53. https://doi.org/10.1039/D3EE00749A.

[17] Chemical Engineering magazine. The Chemical Engineering Plant Cost Index n.d. https://www.chemengonline.com/pci-home/ (accessed December 2, 2024).

[18] Federal Reserve Economic Data (FRED). Employment Cost Index: Wages and Salaries: Private Industry Workers n.d. https://fred.stlouisfed.org/series/ECIWAG (accessed December 2, 2024).

[19] Federal Reserve Economic Data (FRED). Producer Price Index by Commodity: Commercial and Industrial Machinery and Equipment Repair and Maintenance n.d. https://fred.stlouisfed.org/series/WPU551 (accessed December 2, 2024).

[20] Gracida-Alvarez UR, Winjobi O, Sacramento-Rivero JC, Shonnard DR. System Analyses of High-Value Chemicals and Fuels from a Waste High-Density Polyethylene Refinery. Part 1: Conceptual Design and Techno-Economic Assessment. ACS Sustainable Chem Eng 2019;7:18254–66. https://doi.org/10.1021/acssuschemeng.9b04763.

[21] Gao F. Pyrolysis of waste plastics into fuels. PhD Thesis. University of Canterbury. Chemical and Process Engineering, 2010.

[22] Brown JL, Brown RC, Cecon VS, Vorst K, Smith RG, Daugaard TJ. Increasing pyrolysis oil yields and decreasing energy consumption via thermal oxo-degradation of polyolefins. Cell Reports Physical Science 2024;5:101856. https://doi.org/10.1016/j.xcrp.2024.101856.

[23] World Nuclear Association. Heat Values of Various Fuels n.d. https://world-nuclear.org/information-library/facts-and-figures/heat-values-of-various-fuels (accessed December 3, 2024).

[24] Kulas DG, Zolghadr A, Chaudhari US, Shonnard DR. Economic and environmental analysis of plastics pyrolysis after secondary sortation of mixed plastic waste. Journal of Cleaner Production 2023;384:135542. https://doi.org/10.1016/j.jclepro.2022.135542.
[25] Olafasakin O, Ma J, Zavala V, Brown RC, Huber GW, Mba-Wright M. Comparative Techno-economic Analysis and Life Cycle Assessment of Producing High-Value Chemicals and Fuels from Waste Plastic via Conventional Pyrolysis and Thermal Oxo-degradation. Energy Fuels 2023;37:15832–42. https://doi.org/10.1021/acs.energyfuels.3c02321.
[26] Stallkamp C, Hennig M, Volk R, Stapf D, Schultmann F. Pyrolysis of mixed engineering plastics: Economic challenges for automotive plastic waste. Waste Management 2024;176:105–16. https://doi.org/10.1016/j.wasman.2024.01.035.
[27] Fivga A, Dimitriou I. Pyrolysis of plastic waste for production of heavy fuel substitute: A techno-economic assessment. Energy 2018;149:865–74. https://doi.org/10.1016/j.energy.2018.02.094.
[28] Jiang G, Wang J, Al-Salem SultanM, Leeke GA. Molten Solar Salt Pyrolysis of Mixed Plastic Waste: Process Simulation and Technoeconomic Evaluation. Energy Fuels 2020;34:7397–409. https://doi.org/10.1021/acs.energyfuels.0c01052.
[29] Almohamadi H, Alamoudi M, Ahmed U, Shamsuddin R, Smith K. Producing hydrocarbon fuel from the plastic waste: Techno-economic analysis. Korean J Chem Eng 2021;38:2208–16. https://doi.org/10.1007/s11814-021-0876-3.
